# Biologically Aggressive Phenotype and Anti-cancer Immunity Counterbalance in Breast Cancer with High Mutation Rate

**DOI:** 10.1038/s41598-020-58995-4

**Published:** 2020-02-05

**Authors:** Hideo Takahashi, Mariko Asaoka, Li Yan, Omar M. Rashid, Masanori Oshi, Takashi Ishikawa, Masayuki Nagahashi, Kazuaki Takabe

**Affiliations:** 1 Department of Surgical Oncology, Roswell Park Comprehensive Cancer Center, Buffalo, NY USA; 2 Department of Biostatistics and Bioinformatics, Roswell Park Comprehensive Cancer Center, Buffalo, NY USA; 3 Department of Surgical Oncology, Holy Cross Hospital, Trinity Health, Ft Lauderdale, FL USA; 4 Department of Surgery, Massachusetts General Hospital, Boston, MA USA; 5 University of Miami Miller School of Medicine, Miami, FL USA; 6 Department of Breast Surgery and Oncology, Tokyo Medical University, Tokyo, Japan; 7 Department of Surgery, Niigata University Graduate School of Medical and Dental Sciences, Niigata, Japan; 8 Department of Surgery, University at Buffalo Jacobs School of Medicine and Biomedical Sciences, the State University of New York, Buffalo, NY USA; 9 Department of Surgery, Yokohama City University, Yokohama, Japan

**Keywords:** Breast cancer, Surgical oncology, Cancer microenvironment

## Abstract

While cancer cells gain aggressiveness by mutations, abundant mutations release neoantigens, attracting anti-cancer immune cells. We hypothesized that in breast cancer (BC), where mutation is less common, tumors with high mutation rates demonstrate aggressive phenotypes and attract immune cells simultaneously. High mutation rates were defined as the top 10% of the mutation rate, utilizing TCGA and METABRIC transcriptomic data. Mutation rate did not impact survival although high mutation BCs were associated with aggressive clinical features, such as more frequent in ER-negative tumors (p < 0.01), in triple-negative subtype (p = 0.03), and increased *MKI-67* mRNA expression (p < 0.01) in both cohorts. Tumors with high mutation rates were associated with APOBEC3B and homologous recombination deficiency, increasing neoantigen loads (all p < 0.01). Cell proliferation and immune activity pathways were enriched in BCs with high mutation rates. Furthermore, there were higher lymphocytes and M1 macrophage infiltration in high mutation BCs. Additionally, T-cell receptor diversity, cytolytic activity score (CYT), and T-cell exhaustion marker expression were significantly elevated in BCs with high mutation rates (all p < 0.01), indicating strong immunogenicity. In conclusion, enhanced immunity due to neoantigens can be one of possible forces to counterbalance aggressiveness of a high mutation rate, resulting in similar survival rates to low mutation BCs.

## Introduction

Accumulation of somatic mutations, or somatic genome instability, has been the principle of carcinogenesis; cancer cells stem from a clone that has gained the somatically acquired genetic abnormalities, leading to malignant transformation and further progression^1^. With advances in next-generation sequencing technologies, clinical interest in assessing mutation burden and identifying specific mutations in certain cancer types has been growing to utilize targeted therapies or to assess tumor biology, such as FoundationOne genomic panel testing^2–4^.

Mutation rates are variable among cancer types and outliers with significantly high mutation burdens, hypermutation, do exist in many cancer types^5^. Interestingly, the definition of hypermutation has been variable in the literature^5–7^, although the common definition is usually greater than 10–100 mutations per Mega base pairs (Mbps). Campbell and colleagues recently performed comprehensive analysis of hypermutation in various tumor types, providing more insight into tumor evolution and identifying possible clinically actionable mutation signatures^6^. The etiology of hypermutation varies between cancer types; ultraviolet (UV) light is the significant cause of mutations in melanoma, while smoking causes the mutations in non-small cell lung cancer (NSCLC) and head and neck squamous cell carcinoma (SCC)^7,8^. Whereas UV light and smoking are examples of exogenous mutagens, there are endogenous processes for mutation, such as microsatellite instability (MSI), and Apolipoprotein B mRNA editing enzyme catalytic polypeptide-like 3 (APOBEC3) family. MSI is caused by a dysfunctional mismatch repair (MMR) system, resulting in reduction in the length of highly repeated DNA sequences (microsatellites). MSI tumors are found approximately 15% in colorectal cancer and other gastrointestinal cancers, such as gastric cancer and pancreatic cancer, but few in breast cancer^7^. APOBEC family is known for its ability to deaminate genomic DNA cytosines^9^, which normally function in the innate immune system to protect against viral pathogens. However, they can also generate C-to-T (Cytosine to Thymine) mutations in the host genome^10,11^. Recent studies demonstrated that APOBEC3B, a member of APOBEC family, is a mutagenic enzyme and is significantly associated with its mutational load in breast cancer^10,12^.

Recently, it has become clear that increased mutation burden evokes strong immunogenicity in the tumor microenvironment (TME)^5,13^. A large amount of mutations in tumors likely generate abundant neoantigens, recruiting tumor-infiltrating lymphocytes (TILs) in TME that can attack cancer cells. The presence of TILs in TME has been shown to associate with better treatment outcomes^14,15^. It is well known that patients with colorectal cancer with MSI correlated with improved survival, which has been suggested to be a result of increased TILs in TME caused by hypermutation^16^.

Although breast cancer (BC) is known to have low mutation rates^5^, we hypothesized that BCs with high mutation rates would demonstrate biologically worse phenotypes, while simultaneously attracting anti-cancer immune cells in TME.

## Material and Methods

### Data acquisition from TCGA-BRCA and METABRIC

Clinicopathological data for the TCGA- breast invasive carcinoma (BRCA) cases were obtained from the recently released Pan-Cancer Clinical Data Resource^17^ and through cBio Cancer Genomics Portal^18^, as previously described^19–25^. Also, Molecular Taxonomy of Breast Cancer International Consortium (METABRIC) database^26^ was used as a validation cohort in this study. Transcriptomic data of primary tumor samples were analyzed with HTSeq software from Genome Data Commons portal of National Cancer Institute (https://cancergenome.nih.gov/, USA) using TCGA biolinks^27^ Bioconductor package for R (version 2.5.9). The data were normalized using the widely accepted trimmed mean of M-values (TMM) method. Tumor mutation status for specific genes was from the cBioportal, as described previously^19–21^.

The TCGA-BRCA cohort includes 1084 patients, of which 1065 patients were identified to have gene expressions from RNA-sequence, mutation status, and clinicopathological data. The METABRIC cohort contains 1904 patients, of which 1859 patients were identified to have gene expressions from RNA-sequence and clinicopathological data, including mutational status. While the median observation period of the TCGA- BRCA cohort was 26.9 months (Inter-quartile range (IQR): 15–55 months), METABRIC was 112.9 months (IQR: 59–181 months). Given TCGA and METABRIC being de-identified publicly accessible database, institutional Review Board (IRB) was waived.

### Definition of high-mutation BC

Since breast cancers have overall lower mutation rates compared to other cancer types^5^, we used different mutation cutoffs to define BCs with high mutation rate to differentiate from commonly defined hypermutation in the literature. Given no standardized definition of high mutation in BCs in the literature^6,28,29^, we defined high mutation in breast cancer as the top 10% of the whole cohort, which was similar to other studies^8,28^. We rounded this number to 3 mutations/Mbps in TCGA for simplicity. The METABRIC cohort also includes mutation count although the description of mutation count is recorded by count instead of mutation count /Mbps in the TCGA-BRCA cohort. However, with above definition of the top 10% of the whole cohort, we used the cut off of 9 mutation for METABRIC; hence, we were able to use METABRIC database as a validation cohort.

### Analysis of gene expression data

Homologous recombination defect (HRD) score was defined as the unweighted sum of loss of heterozygosity (LOH)^30^, telomeric allelic imbalance (TAI)^31^, and large-scale state transitions (LOS) scores^32,33^. Neoantigen load, the number of peptides predicted to bind with major histocompatibility complex (MHC) proteins, was identified based on HLA types derived from RNA-sequencing data. The counts of neoantigen load were represented as single nucleotide variant (SNV) and Insertion and deletion (Indel) mutations. Values for HRD, neoantigen load (SNV and Indel), and mutation rate (the count of single nucleotide mutation) were collated from the Pan-Cancer Atlas study of Thorsson *et al*.^34^. We also utilized “fraction of genome altered score” calculated on the TCGA BRCA cohort in lieu of copy number variations (CNVs). “Fraction of genome altered score” is the percentage of genome that has been affected by copy number gains or losses^34^.

The Mutant Allele Tumor Heterogeneity (MATH) was calculated to assess tumor heterogeneity and was obtained from the median absolute deviation and the median of its mutant-allele fractions at tumor-specific mutated loci, as described previously^35^.

Measurements of immune activities like T cell receptor diversity and relative fractions in tumors of different types of immune cells were estimated from tumor gene expression with CIBERSORT, a bioinformatic algorithm^34^. Immune cytolytic activity in tumors was defined as the geometric mean of grandzyme A (GZMA) and Perforin 1 (PRF1) expression values in transcripts per million (TPM), as described previously^36,37^. In order to confirm correlation between CYT and grandzyme B (GZMB) or Interferon (IFN)-gamma, Spearman correlation was calculated, which demonstrated r = 0.819 (p < 0.01) and r = 0.789 (p < 0.01), respectively (Supplementary Fig.S1). Additionally, as METABRIC transcriptome was derived from the microarray, we used the geometric mean of GZMA and PRF1 to estimate the cytolytic activity in the validation cohort.

### Gene Set Enrichment Analysis (GSEA)

GSEA was performed comparing the high mutation and low mutation tumors, utilizing the Hallmark gene sets^38^ with the software provided by the Broad Institute (https://software.broadinstitute.org/gsea/index.jsp), as described before^20,21,24,39^.

### Statistical analysis

Statistical analyses were performed using R software (version 3.6, http:///www.r-project.org/) Bioconductor (http://bioconductor.org/) and Prism (version 7.0d; GraphPad Software®). Progression-free interval (PFI) was defined as the time between date of diagnosis and the date of progression of BC, disease-free interval (DFI) as the time between date of diagnosis and the date of diagnosis of a recurrent BC, disease-specific survival (DSS) as the time from date of diagnosis to the date of death by BC, and overall survival (OS) as the time from date of diagnosis to the date of death by any cause. For survival analyses, Kaplan-Meier method with log-rank test was performed with greyzoneSurv (version 1.0) packages in R. The differences between the two groups were assessed using Fisher’s exact were used for categorical variables. A two-sided p value < 0.05 was considered statistically significant.

## Results

### Patients with high mutation tumors had similar survival compared to those with low mutation tumors

**Figure 1:**
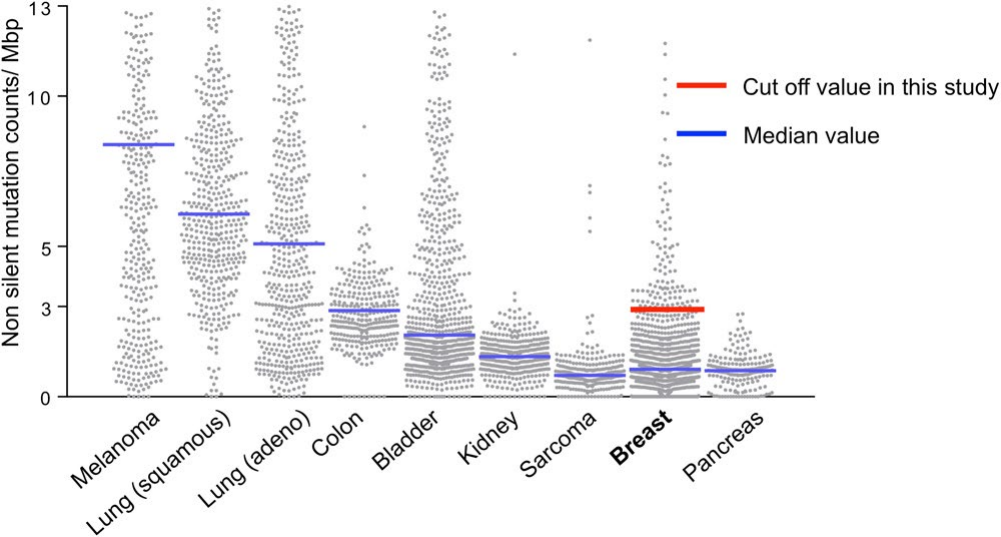
Non-silent mutation count distribution in TCGA multiple cancer cohorts, the top 10% of the non-silent mutation counts, rounded to 3/Mbps (red bar), is defined as BCs with high mutation rates in the present study. Blue bar represents the median values. TCGA, The Cancer Genome Atlas; Mbps, Mega base pairs; BC, breast cancer.

The majority of patients with breast cancer revealed far less mutation burden compared to melanoma, lung cancer, bladder cancer, or colon cancer in TCGA cohort in agreement with previous reports that analyzed different cohorts (Fig. 1)^40,41^. With our definition of high mutation rate in BCs, 114 patients (10.7%) had high mutation rates and 951 patients (89.3%) had low mutation rates in the TCGA training cohort. Similarly, in the validation cohort, 203 patients (10.9%) had high mutation rates and 1656 (89.1%) had low mutation rates. Commonly mutated genes in the TCGA cohort were shown in Supplementary Table S1.

**Figure 2:**
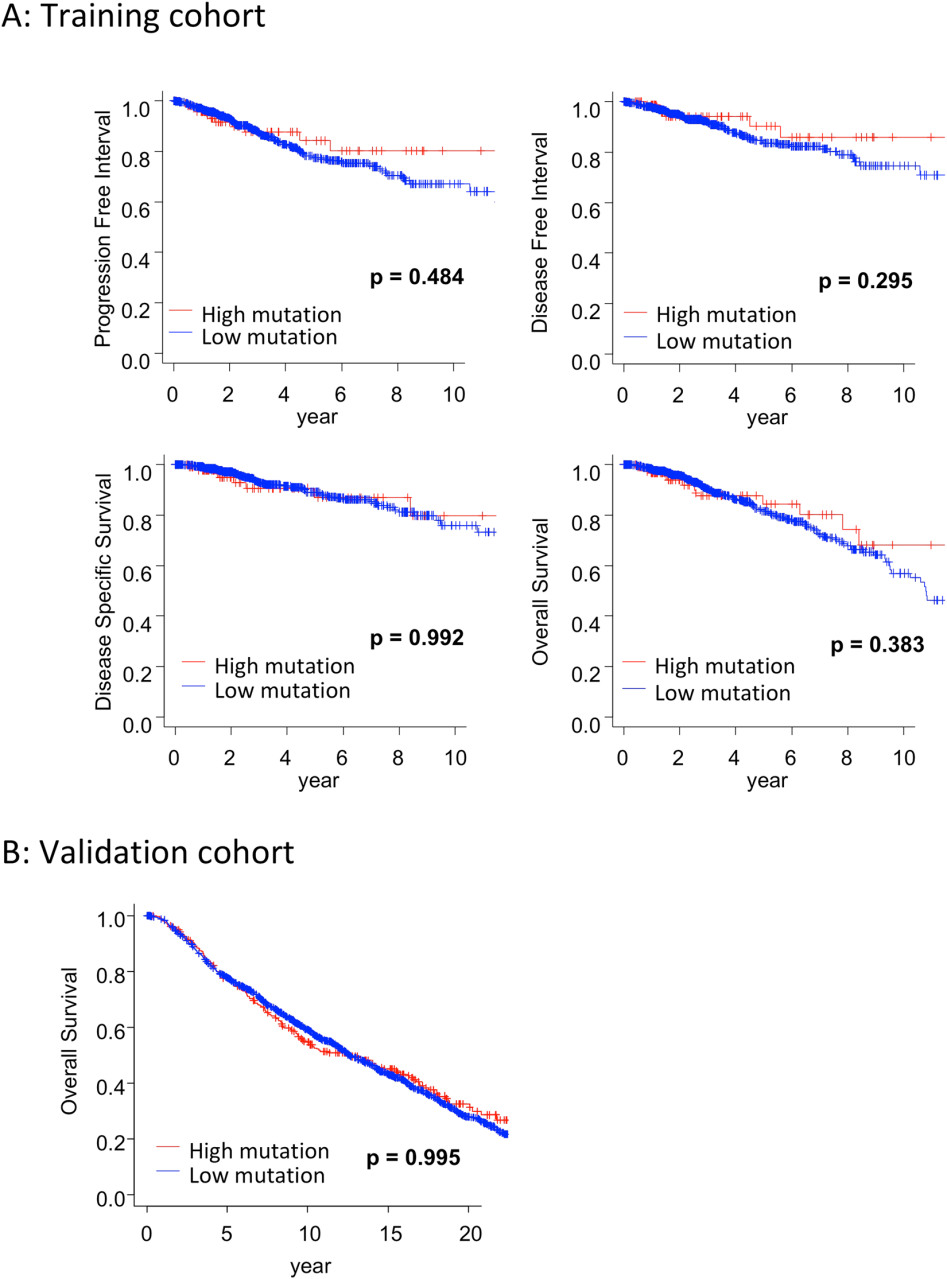
(**A**) Kaplan-Meier curves depicting PFI, DFI, DSS, and OS by mutation rates, using the training cohort. (**B**) Kaplan-Meier curves depicting OS by mutation rates, using the validation cohort. No difference in clinical outcomes based on tumor mutation rates alone. PFI, progression free interval; DFI, disease free interval; DSS, disease specific survival; OS, overall survival.

We first hypothesized that BCs with high mutation rates demonstrate aggressive phenotypes resulting in worse survival compared to low mutation BCs. However, contrary to our hypothesis, there was no significant difference in PFI (p = 0.484) and DFI (p = 0.295) between BCs with high mutation rates and BCs with low mutation rates. Furthermore, there was no significant difference in DSS (p = 0.992) and OS (p = 0.383) between the two groups (Fig. 2A). OS in the validation cohort did not demonstrate any difference as well (p = 0.995; Fig. 2B). Hence, our hypothesis was rejected by these survival analyses.

### BCs with high mutation rates did possess aggressive clinical features compared to low mutation BCs

**Figure 3:**
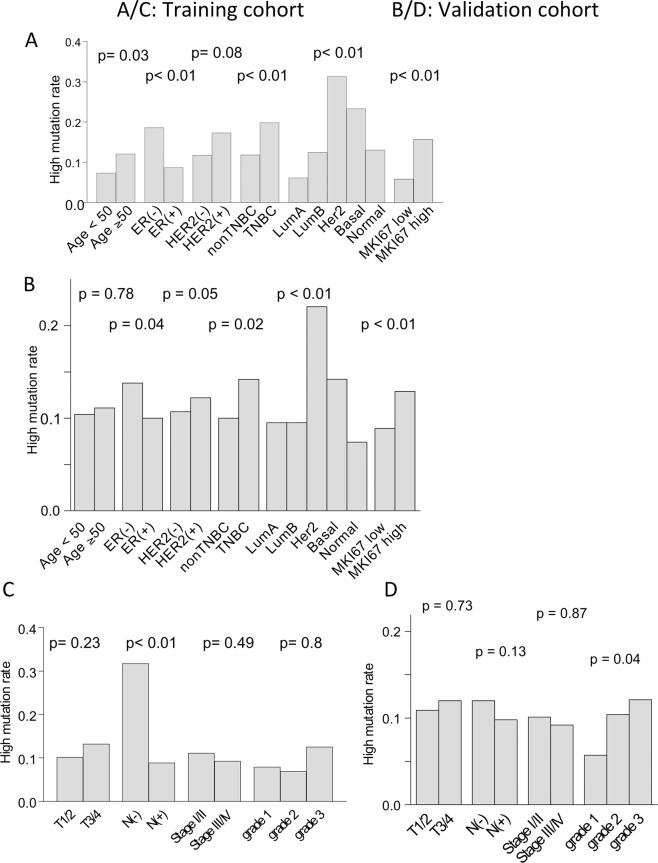
(**A**) Tumors with high mutation rates were more common in patients with age ≥50 (p = 0.03), ER (−) (p < 0.01), and TNBC (p < 0.01). Also, tumors with high mutation rates were more often in Luminal B, Her2, and Basal subtypes, compared to Luminal A subtype on PAM50 classification. Furthermore, tumors with high mutation rates demonstrated higher gene expression of *MKI*-67 (p < 0.01). (**B**) Similar results were noted in the validation cohort; higher mutation rates in ER (−) tumors (p = 0.04), TNBC tumors (p = 0.02), higher proportions in HER2 and Basal subtypes compared to Luminal A subtype on PAM50 classification (p < 0.01), and higher gene expression of *MKI-67* (p < 0.01). (**C**) In the training cohort, tumors with high mutation rates were significantly associated with negative node status (p < 0.01), but not with AJCC T category (p = 0.23), pathological stage (p = 0.49), or histological grade (p = 0.8). (**D**) No difference in mutation rates in AJCC T (p = 0.73), N category (p = 0.13), or pathological stage (p = 0.87) in the validation cohort. Higher mutation rate was significantly more in the grade 3 tumors (p = 0.04). ER, estrogen receptor; TNBC, triple negative breast cancer; AJCC, American Joint Committee for Cancer.

Although there was no difference in survival rate based on the tumor mutation burden, we speculated that BCs with high mutation rates would possess aggressive clinical features. High mutation rate was found to be more frequent in patients with age ≥50 compared to patients with age <50 (p = 0.03), in Estrogen receptor (ER)-negative tumors (p < 0.001), and in triple-negative tumors (p = 0.03). A significant difference was also observed in PAM50 classification^42^ as well; high mutation rate was more common in Luminal B, Her2, and Basal types, compared to Luminal A subtype (p < 0.01; Fig. 3A, Supplementary Table S2), all known to be aggressive subtypes. There were no significant differences in tumor characteristics among different subtypes as well (Supplementary Table S3). Furthermore, tumors with high mutation rate demonstrated higher gene expression of *MKI-67* (p < 0.001), reflecting higher proliferation ability. In the validation cohort, similar trend was observed, such as high mutation rate in ER negative tumors (p = 0.04), triple-negative tumors (p = 0.02), in higher MKI-67 expression (p < 0.01), as well as similar rate in PAM50 classification (p < 0.01) (Fig. 3B, Supplementary Table S4).

However, interestingly, this aggressiveness of tumors with high mutation rate did not reflect tumor size or pathological stage (Fig. 3C, D, Supplementary Tables S2,S3). We also noted different results between two cohorts; higher mutation rate in lymph node negative group in the training cohort and higher mutation rate in the higher grade BCs in the validation cohort. With these findings, we suspected that aggressive clinical features of BCs with high mutation rates might be mitigated by other protective mechanisms.

### Mutation sources and Neoantigen loads in BCs with high mutation rates

**Figure 4:**
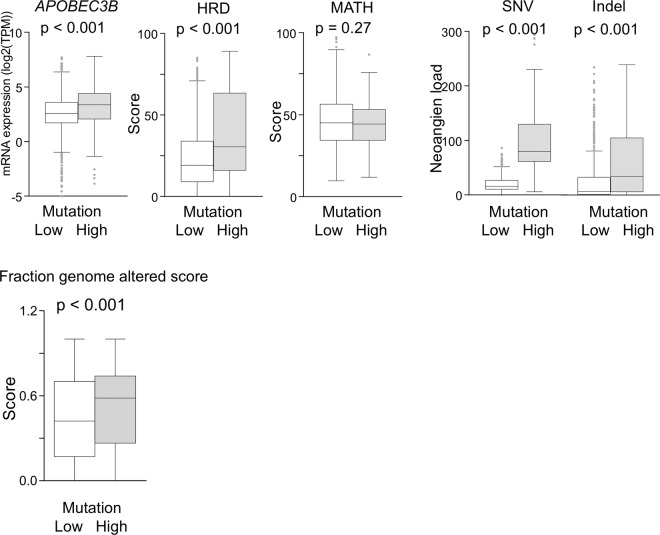
Tumors with high mutation rates were derived from not only APOBEC3B (p < 0.001), but also HRD (p < 0.001). Despite multiple mutation sources in the high mutation rate group, heterogeneity measured by MATH score (p = 0.27) was similar between two groups. Tumors with high mutation were associated with increased neoantigen loads, represented by SNV and Indel (p < 0.001, respectively). Fraction genome altered score was significantly elevated in tumors with high mutation rate. APOBEC3B, Apolipoprotein B mRNA editing enzyme catalytic polypeptide-like 3B; HRD, homologous recombination; MATH, Mutant Allele Tumor Heterogeneity; SNV, single nucleotide variant; Indel, Insertion and deletion.

Based upon previous reports, we hypothesized that APOBEC3B, homologous recombination defect (HRD), and intra-tumoral heterogeneity are possible sources of mutation in BCs with high mutation rates. Indeed, gene expression of APOBEC3B, a known strong DNA mutator in BCs^12^, was significantly elevated in BCs with high mutation rates (p < 0.001; Fig. 4). Double-stranded DNA damages are usually repaired with homologous recombination, as it is more efficient than the non-homologous method^33,43^. Therefore, HRD leads to increased DNA mutation in the tumor. BCs with high mutation rates demonstrated higher HRD scores (p < 0.001; Fig. 4), which suggested that HRD is also a possible mutagen in addition to APOBEC3B in BCs. Although there were multiple other sources of mutation in BCs with high mutation rates, such as age-related deterioration, tumor heterogeneity measured by MATH score was not significantly different (p = 0.27; Fig. 4).

Furthermore, cancer cells with large numbers of mutations are known to generate neoantigens; thus, we investigated the neoantigen loads in BCs with high mutation rates, which were calculated by two different methods, SNV and Indel. We found that increased mutation burdens in the tumor were associated with increased neoantigen loads (p < 0.001; Fig. 4), which suggested increased immunogenicity against BCs with high mutation rates. A high burden of copy number variations (CNVs) is known to decrease the tumor aggressiveness possibly from the attracted immune cells^44^. Although our result did not demonstrate survival benefit as shown in Fig. 2, the fraction genome altered score (used in lieu of CNVs) was significantly elevated in BCs with high mutation rates (p < 0.001, Fig. 4).

### Gene set enrichment analysis (GSEA) revealed that gene sets related to cell proliferation and immune activity were enriched in BCs with high mutation rates

Despite aggressive biological characteristics in BCs with high mutation rates, survival did not correlate with mutation burden. Since neoantigen loads were elevated in the tumors with high mutation rates, we further hypothesized that enhanced immunity might mitigate the aggressiveness of BCs with high mutation rates and thus explain the survival findings. Therefore, we performed GSEA to test this hypothesis.

**Figure 5:**
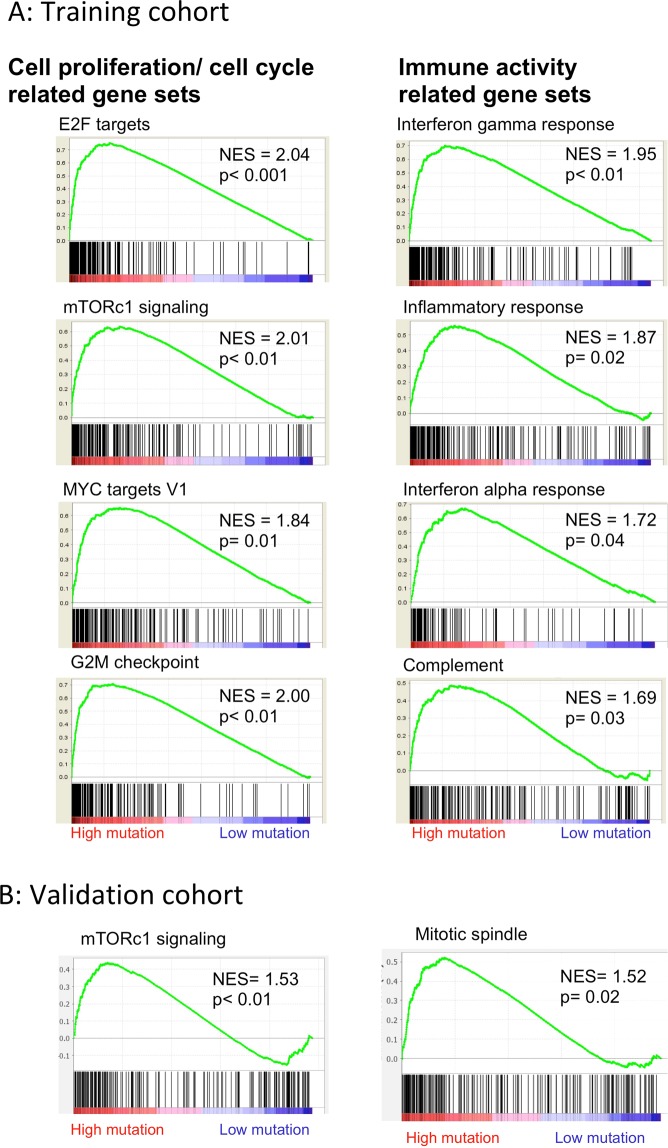
(**A**) Enrichment plots of tumors with high mutation rates by gene sets enrichment analysis (GSEA) in the training cohort. Tumors with high mutation rates were associated with cell proliferation/ cell cycle related gene sets, such as E2F targets (NES = 2.04, p < 0.001), mTORc1 signaling (NES = 2.01, p < 0.01), MYC targets v1 (NES = 1.84, p = 0.01), and G2M checkpoint (NES = 2.00, p < 0.01). Furthermore, tumors with high mutation rates were associated with immune activity related gene sets, such as Interferon gamma response (NES = 1.95, p < 0.01), Inflammatory response (NES = 1.87, p = 0.02), Interferon alfa response (NES = 1.72, p = 0.04), and Complement (NES = 1.69, p = 0.03). (**B**) Enrichment plots of tumors with high mutation rates by gene sets enrichment analysis (GSEA) in the validation cohort. Tumors with high mutation rates were associated with only cell proliferation/ cell cycle related gene sets, mTORc1 signaling (NES = 1.53, p < 0.01), and Mitotic spindle (NES = 1.52, p = 0.02). NES, normalized enrichment score.

BCs with high mutation rates enriched cell proliferation and cell cycle related gene sets; E2F targets (Normalized enrichment score (NES) = 2.04, p < 0.001), mTORc1 signaling (NES = 2.01, p < 0.01), MYC targets (NES = 1.84, p = 0.01), and G2M checkpoint (NES = 2.00, p < 0.01), which suggest aggressive cancer phenotypes (Fig. 5A). This was confirmed in the validation cohort as well (mTORc1 signaling (NES = 1.53, p < 0.01) and mitotic spindle (NES = 1.52, p = 0.02)) (Fig. 5B). On the other hand, immune activity related gene sets, including Interferon gamma response (NES = 1.95, p < 0.01), Inflammatory response (NES = 1.87, p = 0.02), Interferon alpha response (NES = 1.72, p = 0.04), and Complement (NES = 1.69, p = 0.03) were also enriched, suggesting enhanced immunity in BCs with high mutation rates (Fig. 5A). To our surprise, none of immune activity related gene sets were enriched in the validation cohort. Taken together, based on the transcriptomic profiles, we speculated that BCs with high mutation rates demonstrated aggressive phenotype and simultaneously provoked enhanced immune activity.

Contrary to BCs with high mutation rate, BCs with low mutation rate were enriched with both early and late estrogen response gene sets (Supplementary Fig.S2), which support the above findings that less mutation was found in ER positive BCs.

### BCs with high mutation rates revealed significantly increased anti-cancer immune cell infiltrations and were associated with higher T cells exhaustion markers

**Figure 6:**
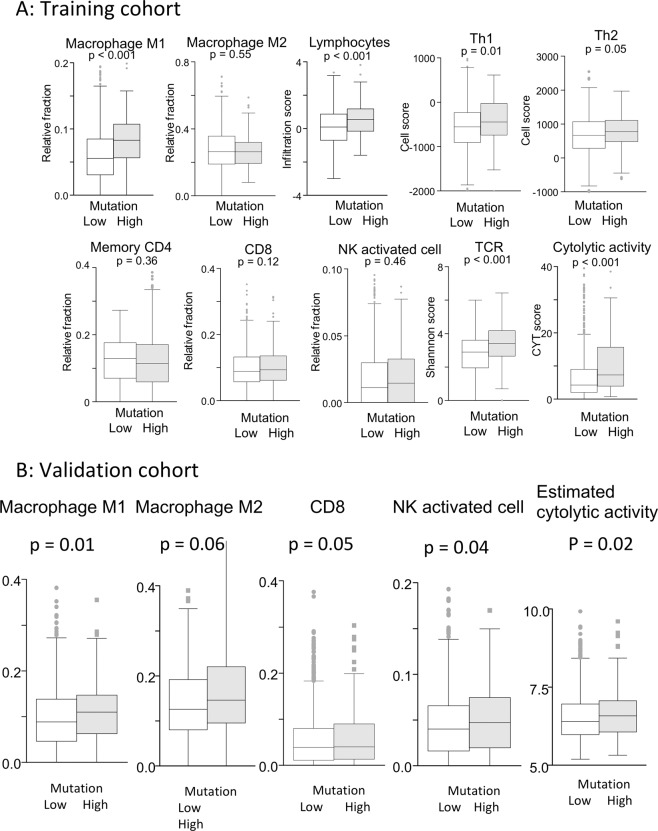
(**A**) In the training cohort, CIBERSORT demonstrated that high infiltration of Macrophage M1 (p < 0.001), but not M2 (p = 0.55) in tumors with high mutation rates. Similarly, infiltration score of lymphocytes (p < 0.001) was elevated, especially Th1 (p = 0.01), but not Th2 (p = 0.05) or Memory CD4 T cells (p = 0.32). Neither CD8 + T cells (p = 0.12) nor NK cells (p = 0.46) were significantly infiltrated in tumors with high mutation rates. Additionally, tumors with high mutation rates correlated with significantly more TCR diversity (p < 0.001), reflecting higher neoantigen load in tumors. With this increased TCR diversity, CYT (p < 0.001) was also significantly elevated in tumors with high mutation rates. (**B**) Similar trend was observed in the validation cohort. Tumors with high mutation rates were associated with higher infiltration of Macrophage M1 (p = 0.01), but not M2 (p = 0.06) in tumors with high mutation rates. CD8 + T cells (p = 0.05) and NK cells (p = 0.04) were significantly infiltrated in tumors with high mutation rates. Tumors with high mutation rates was associated with estimated cytolytic activity (p = 0.02). Th1, helper T lymphocytes; Th2, helper T lymphocytes; NK cells, natural killer cells; TCR, T cell receptors; CYT, cytolytic activity score;.

Given strong immunogenicity in BCs with high mutation rates in the training cohort, we hypothesized that they would also a have higher infiltration of cytotoxic lymphocytes in the TME. Immune cell composition in TME was estimated by CIBERSORT, which revealed higher rates of anti-cancer M1 macrophage (p < 0.001), but not pro-cancer M2 macrophage (p = 0.55), and increased infiltration of lymphocytes (p < 0.001), especially anti-cancer helper T cell 1 (Th1, p = 0.01). Th2 (p = 0.05) or Memory CD4 T cells (p = 0.36) were not significantly increased (Fig. 6A). Similar trends were observed in the validation cohort, such as higher rate in M1 macrophage but not in M2 macrophage (Fig. 6B). Interestingly, composition in Natural Killer (NK) cells and CD8 + T cells, which are known major players in anti-cancer immunity, were found to be different between two cohorts (Fig. 6A,B). Additionally, BCs with high mutation rates were associated with significantly more T cell receptor (TCR) diversity (p < 0.001), reflecting more neoantigen loads in tumors. Consistent with this increased TCR diversity, cytolytic activity score (CYT) (p < 0.001; Fig. 6A) was also significantly elevated in tumors with high mutation rate. Cytolytic activity in validation cohort was also elevated in BCs with high mutation rates as well (p = 0.02; Fig. 6B).

**Figure 7:**
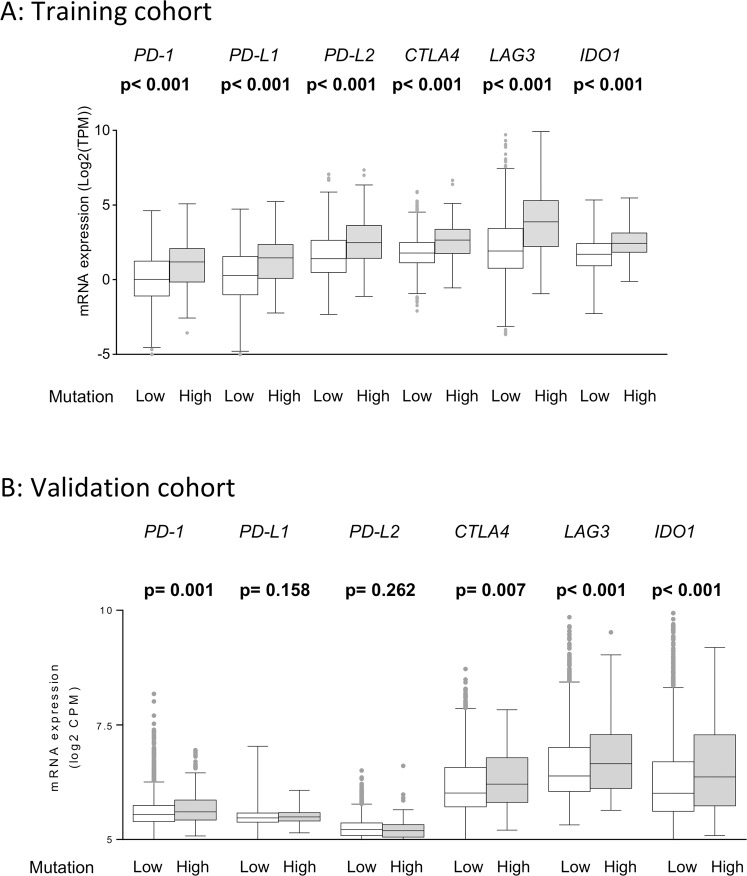
(**A**) High mutation rates correlated with significantly higher expression of T cell exhaustion markers, including PD-1, PD-L1, PD-L2, CTLA4, LAG3, and IDO1 (all p < 0.001) in the training cohort. (**B**) High mutation rates were similarly associated with significantly higher expression of PD-1 (p = 0.001), CTLA4 (p = 0.007), LAG3 (p < 0.001), and IDO1 (p < 0.001) in the validation cohort. PD-1, programmed cell death 1; PD-L1, programmed death ligand 1; PD-L2, programmed death ligand 2; cytotoxic T-lymphocyte-associated antigen 4, CTLA4, LAG3, lymphocyte activating 3; IDO1, indoleamine 2,3-dioxygenase 1.

Lastly, we speculated that T cell exhaustion markers would be increased as a negative feedback loop for the strong immunogenicity in high mutation rate BC tumors. We found that the expression of major T cell exhaustion markers; including programmed cell death 1 (PD-1), PD-L1, programmed death ligand 2 (PD-L2), cytotoxic T-lymphocyte-associated antigen 4 (CTLA4), lymphocyte activating 3 (LAG3), and indoleamine 2,3-dioxygenase 1 (IDO1), were significantly elevated in BCs with high mutation rates (all p < 0.001) (Fig. 7A). Similar results in PD-1 (p = 0.001), CTLA4 (p = 0.007), LAG3 (p < 0.001), and IDO1 (p < 0.001) were observed in the validation cohort (Fig. 7B).

These results suggest that the aggressive phenotype of BCs with high mutation rates was counterbalanced by stronger immunogenicity, thus resulting in no difference in clinical outcomes based on differences in BC mutation rates alone.

## Discussions

In order to elucidate the effect of mutation rate in breast cancer, where mutation is far less common compared to other cancer types, we analyzed 2,924 patients from TCGA and METABRIC. BCs with high mutation rates, defined as the top 10% of the non-silent mutation count in the cohort, were found more often in triple-negative breast cancers (TNBCs) and hormone receptor negative tumors. Tumors with high mutation rates demonstrated aggressive clinical features, such as elevated *MKI-67* expression and enriched cell cycle and cell proliferation related gene sets on GSEA. Despite these aggressive phenotypes, clinical outcomes including PFI, DFI, DSS, and OS in patients with BCs with high mutation rates were not significantly different compared to those with low mutation rates. To explain these seemingly contradictory results, we also found that BCs with high mutation rates were associated with a significant enrichment of immune activity related gene sets, such as Interferon gamma response and Interferon alpha response on GSEA in the training cohort. Additionally, BCs with high mutation rates demonstrated higher CYT and increased anti-cancer immune cells infiltration such as M1 macrophage and Th1 in both cohorts. Therefore, our results suggested that the phenotypic aggressiveness of tumors with high mutation rates was counterbalanced by enhanced anti-cancer immunity, leading to the absence of any significant differences in clinical outcome by mutation rate alone.

Carcinogenesis and cancer progression are known to be driven by the accumulation of somatic mutations in cancer cells^1^. Cancer cells gain more phenotypic aggressiveness with mutations. Angus *et al*. have demonstrated that the median tumor mutation burden was significantly higher than the primary BC, and high mutation burden was not associated with BC subtype in metastatic BC^45^. Taken together with our result, the mutation profiles were often significantly different between the primary tumors and metastatic sites^45–47^. Increased diversity of mutation may lead to the selection of aggressive subclones even without treatment effect induced selection pressures^48^. Mutations in certain genes may alter tumor biology to more aggressive phenotypes, such as KRAS or BRAF mutation in colorectal cancer^49^. While the current study suggests that increased mutation rate in BC is associated with its biological aggressiveness, McFarland *et al*. demonstrated increased passenger mutation reduce proliferative fitness of BC, tumor growth, and metastatic progression^50^. Accumulation of passenger mutations may have deleterious effect to cancer on its own in addition to immunogenic role, which was observed in the present study.

Mutation rates are known to significantly vary among different cancer types^6^. As demonstrated in Fig. 1, mutation rates in breast cancer, pancreatic cancer, or sarcoma were significantly lower compared to melanoma, lung cancer, and bladder cancer in TCGA, which is consistent with other cohorts. It has been speculated that this variability in mutation rate is likely due to the differences in the etiology of mutagenesis^2^. Multiple exogenous and endogenous mutagens have been identified in various cancer types, such as UV light in melanoma, smoking in NSCLC and head and neck SCC, Epstein-Barr (EB) virus infection in head and neck SCC, and MSI in colon cancer, gastric cancer, and endometrial cancer^7^. Mutation rates in breast cancer are significantly less frequent because endogenous or exogenous mutagens are rarely involved in its mutagenesis^5^. On the other hand, APOBEC3B^12^ and HRD^33^ are known to generate mutation in BCs and the tumors with these mutagens are more likely to have higher mutation rates, which was the case in the current study, although there are other possible etiologies as well, such as age-related deterioration and previous treatment^45^. Pretreatment and subsequent induced selection pressures increase tumor mutation burden in breast cancers^45^. However, two cohorts used consist of treatment naïve patients; hence, pretreatment with induced selection pressure is unlikely to be the cause of mutation rates in the present study. BRCA1/2 mutation status is also known to increase mutation burden in BCs, and this was investigated in the present study. Unfortunately, the TCGA cohort has only 57 patients with known BRCA status. We found no difference between high mutation and low mutation groups in terms of mutation rates although this is likely due to the small sample size (data not shown). Furthermore, the METABRIC cohort does not include BRCA status as well.

Several studies demonstrated that hypermutation is associated with increased TILs in TME due to neoantigens generation^5,7,13^. Innocenti and colleagues recently reported that higher tumor mutation rates correlated with better survival in patients with microsatellite stable (MSS) colorectal cancer, where mutation rates are far less common than MSI-high tumor^51^. They suggested that the survival benefit was a reflection of increased neoantigens and increased TILs induced by the higher mutation rate. This correlation between high mutation load and immunogenic neoantigens load have been described in meta-analysis from six cancer types^52^. Similarly, increased TILs in TME were associated with increased response to neoadjuvant chemotherapy, higher complete clinical response rate, and subsequent longer survival in TNBCs and HER2 positive BCs, in which higher mutation rates are often identified^5,53^. Our results revealed that BCs with high mutation rates, although the mutation rate was far lower than in other cancer types, have attracted effective immune cells in their microenvironment, such as macrophage M1 and CD4 + T cells. Interestingly, although the number of effector cells, such as CD8 + T cells or NK cells, was inconclusive between the two cohorts, CYT, representing function of tumor killing, is enhanced in BCs with high mutation rates in both cohorts. This finding is in agreement with the notion that the presence of functioning CD8 + T cells is more important for immunogenicity, rather than the mere number of T cells in TME. Similarly, as demonstrated in melanoma, the balance between effector cells and suppressor cells might be more important than the actual number of lymphocytes^54^.

Impassion130 trial^55^, which resulted in FDA approval of anti-PD-L1 antibody for TNBCs, demonstrated a significantly improved objective response rate, median progression-free survival, and OS in the PD-L1 positive cohort, similar to other cancer types^56–58^. Although positive PD-L1 expression is a current pre-requisite for anti-PD-L1 therapy in breast cancer, the utility of PD-L1 expression as an ideal biomarker for checkpoint inhibition remains equivocal given mixed results from multiple trials with ICIs regardless of PD-L1 expression^59–62^. While high mutation rates have been considered as a promising biomarker for ICIs in other cancer types^63^, there is limited evidence in BCs, given overall low mutation rates. The results in the present study suggest that BCs with high mutation rates demonstrate enhanced anti-cancer immunity, resulting in a counterbalancing of the aggressive phenotypes. Furthermore, T cell exhaustion markers were significantly expressed as a negative feedback loop for increased immune cell infiltration and enhanced anti-cancer immunity. Hence, as Atezolizumab was approved for TNBCs with positive PD-L1 expression recently^55^, it is conceivable that ICIs would be efficacious in BCs with high mutation rates, regardless of subtypes. Furthermore, with blocking the negative feedback of T cells in BCs with high mutation rates, it might be possible to improve clinical outcomes with a combination of two or more immune checkpoint inhibitors, which several ongoing clinical trials would be able to address in the near future.

There are a few limitations in the present study. Despite its significant benefits with clinicopathological and excellent survival data along with gene expression information, TCGA has a few disadvantages, such as short follow up duration. As 3-year survival rate of breast cancer is approximately 95%, our prognostic analysis with the training cohort could be immature given the short median follow up. To overcome this shortcoming, this survival result was validated by the METABRIC cohort that has twice as many patients as the TCGA cohort and significantly longer follow-up period. Lastly, this study was based on the gene expression profiles of surgically resected primary tumors only; thus, the role of mutation count is unclear in metastatic sites. Lastly, this study does not include any *in-vitro* or *in-vivo* experiments; therefore, all our findings are based exclusively on correlation. In order to further investigate the effect of high mutation rate in breast cancer, the experimental approach will be required.

## Conclusion

In conclusion, breast cancer with high mutation rates demonstrated similar clinical outcomes compared to the low mutation group, even though the high mutation rate group possessed more aggressive phenotypes. APOBEC3B and HRD were associated with BCs with high mutation rates, partly contributing to mutation generation in addition to other mutagenic etiologies, such as age-related deterioration. Additionally, high mutation rate BCs appeared to be associated with increased neoantigen loads and enhanced anti-cancer immunity. The increased anti-cancer immune activity could be one of the forces that counterbalanced the aggressiveness of BCs with high mutation rates. Given its enhanced immunogenicity, immune checkpoint inhibition may be indicated in this subset of patient population.

## Supplementary information

Supplementary information.
