# Supplementary material for: Biologically Aggressive Phenotype and Anti-cancer Immunity Counterbalance in Breast Cancer with High Mutation Rate
Source: Sci Rep. 2020 Feb 5;10:1852. doi: 10.1038/s41598-020-58995-4 (PMC7002588; doi:10.1038/s41598-020-58995-4)
Supplement: Supplementary file 1 — Supplementary information. [file 41598_2020_58995_MOESM1_ESM.docx]

Biologically Aggressive Phenotype and Anti-cancer Immunity Counterbalance in Breast Cancer with High Mutation Rate

Hideo Takahashi*^1^, Mariko Asaoka*^1^, Li Yan^2^, Omar M. Rashid^3-5^, Masanori Oshi^1^, Takashi Ishikawa^6^, Masayuki Nagahashi^7^, Kazuaki Takabe^1, 6-9^

* These authors contributed equally.

1: Department of Surgical Oncology, Roswell Park Comprehensive Cancer Center, Buffalo, NY, USA.

2: Department of Biostatistics and Bioinformatics, Roswell Park Comprehensive Cancer Center, Buffalo, NY, USA.

3: Department of Surgical Oncology, Holy Cross Hospital, Trinity Health, Ft Lauderdale, FL, USA

4: Department of Surgery, Massachusetts General Hospital, Boston, FL, USA

5: University of Miami Miller School of Medicine, Miami, FL, USA

6: Department of Breast Surgery and Oncology, Tokyo Medical University, Tokyo, Japan.

7: Department of Surgery, Niigata University Graduate School of Medical and Dental Sciences, Niigata, Japan.

8: Department of Surgery, University at Buffalo Jacobs School of Medicine and Biomedical Sciences, the State University of New York, Buffalo, NY, USA.

9: Department of Surgery, Yokohama City University, Yokohama, Japan.

**Corresponding author:**

Kazuaki Takabe, MD, PhD, FACS

Department of Surgical Oncology

Roswell Park Comprehensive Cancer Center

Elm & Carlton Streets

Buffalo, NY 14263 USA

Phone: 716-845-2918

Fax- 716-845-1668

Email: kazuaki.takabe@roswellpark.org

**Supplement Figures**

**
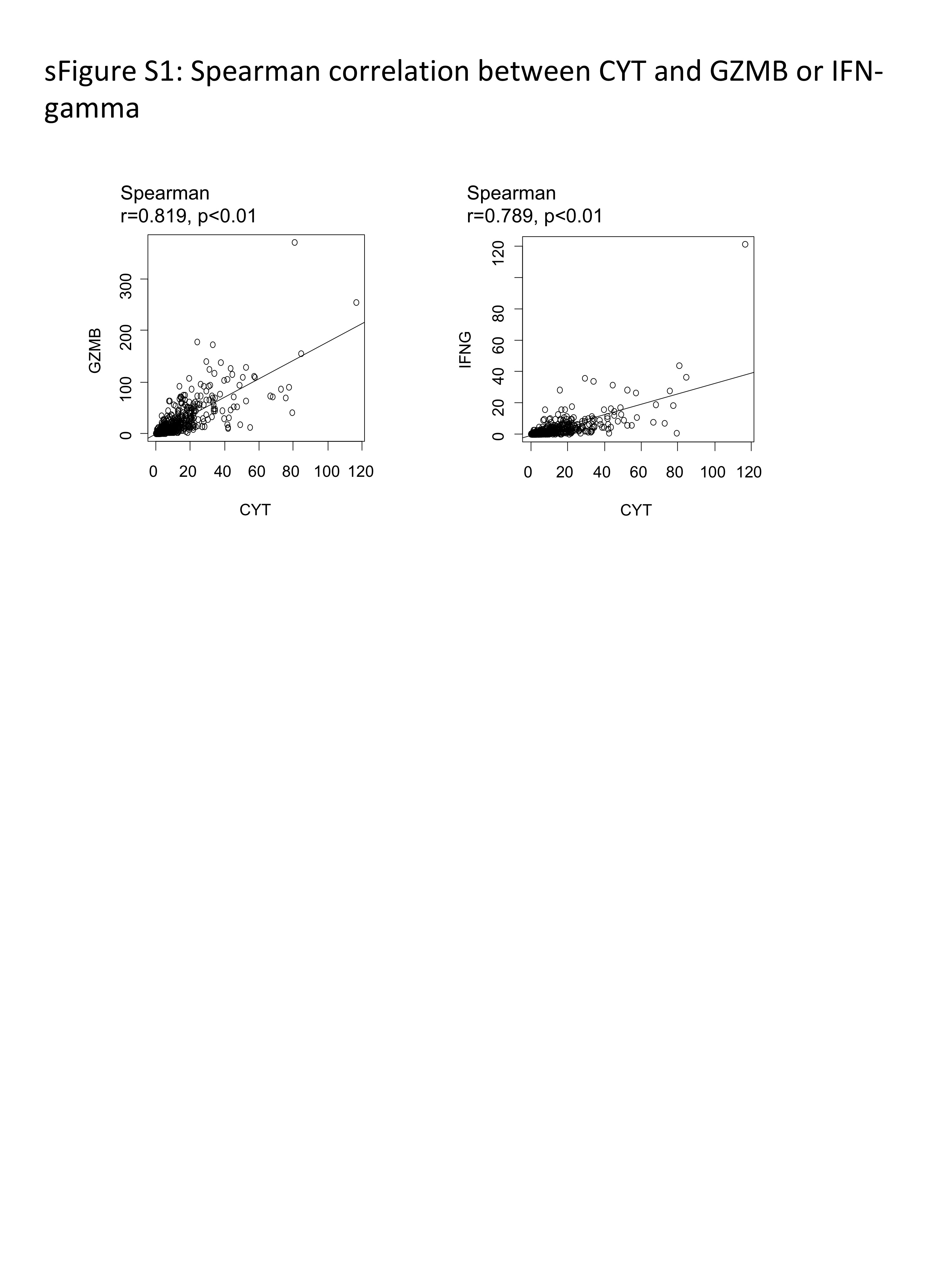
**

**
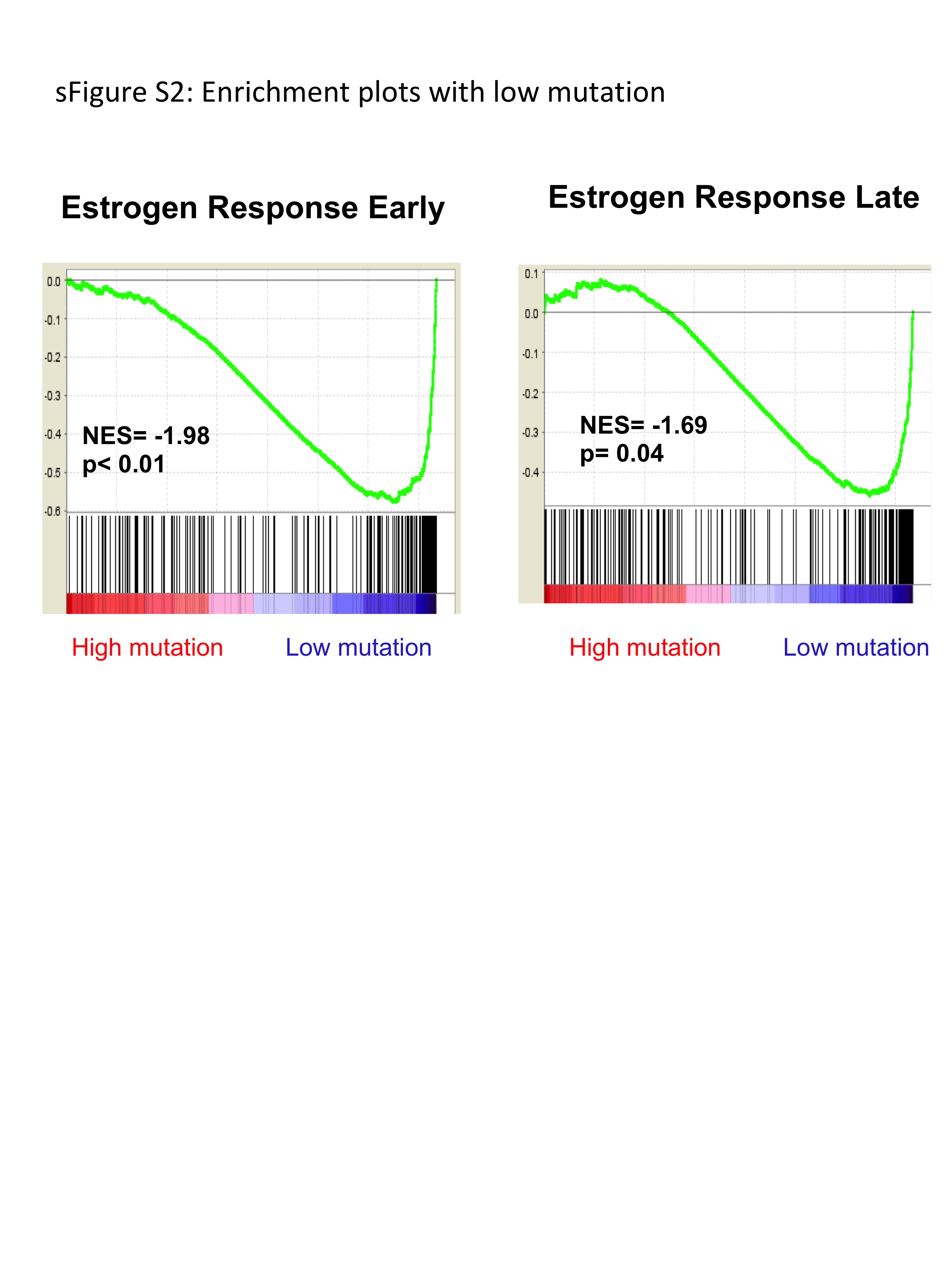
**

**Supplementary Fig. S1** Spearman correlation between CYT and GZMB and IFN-Gamma; r=0.819 (p<0.01) and r=0.789 (p<0.01), respectively

CYT, cytolytic activity score; GZMB, grandzyme B; IFN, Interferon

**Supplementary Fig. S2** Enrichment plots of tumors with low mutation rates by GSEA in the training cohort. Tumors with low mutation rates enriched Estrogen Response Early (NES= -1.98, p<0.01) and Late (NES= - 1.69, p=0.04) gene sets.

NES, normalized enrichment score

**Supplementary Tables**

Supplementary Table S1: Commonly mutated genes in the training cohort

|  | **All tumor with gene mutations** | | **Gene mutation in high mutation tumors (N)** | | **Gene mutation in low mutation tumors (N)** | | **p value** |
| --- | --- | --- | --- | --- | --- | --- | --- |
|  | N | % | + | - | + | - |  |
| PIK3CA | 311 | 32.9% | 41 | 65 | 270 | 569 | 0.19 |
| TP53 | 291 | 30.8% | 49 | 57 | 242 | 597 | < 0.001 |
| TTN | 147 | 15.6% | 49 | 57 | 98 | 741 | < 0.001 |
| CDH1 | 109 | 11.5% | 18 | 88 | 91 | 748 | 0.07 |
| GATA3 | 96 | 10.2% | 7 | 99 | 89 | 750 | 0.23 |
| MUC16 | 74 | 7.8% | 27 | 79 | 47 | 792 | < 0.001 |
| MAP3K1 | 70 | 7.4% | 7 | 99 | 63 | 776 | 0.85 |
| KMT2C | 66 | 7.0% | 12 | 94 | 54 | 785 | 0.07 |
| MUC12 | 53 | 5.6% | 11 | 95 | 42 | 797 | 0.04 |
| MUC4 | 52 | 5.5% | 10 | 96 | 42 | 797 | 0.07 |

Supplementary Table S2: Training cohort patient characteristics

|  |  | **High mutation**  **(N)** | **Low mutation**  **(N)** | **p value** |
| --- | --- | --- | --- | --- |
| Age | ≥ 50 | 93 | 675 | 0.03 |
|  | < 50 | 21 | 264 |  |
| AJCC T | T 1/2 | 90 | 798 | 0.23 |
|  | T 3/4 | 23 | 151 |  |
| AJCC N | N 0 | 65 | 410 | < 0.01 |
|  | N + | 48 | 539 |  |
| pStage | Stage 1/2 | 86 | 686 | 0.485 |
|  | Stage 3/4 | 24 | 234 |  |
| ER | - | 43 | 188 | < 0.01 |
|  | + | 68 | 712 |  |
| PR | - | 62 | 271 | < 0.01 |
|  | + | 49 | 626 |  |
| HER2 | - | 64 | 479 | 0.079 |
|  | + | 27 | 129 |  |
| TNBC | No | 69 | 514 | < 0.01 |
|  | Yes | 22 | 89 |  |
| *MKI67* | High | 83 | 446 | < 0.01 |
|  | Low | 31 | 498 |  |

AJCC, American Joint Committee for Cancer; ER, estrogen receptor; PR, progesterone receptor; TNBC, triple negative breast cancer

Supplementary Table S3: Tumor characteristics among PAM50 classification (Training cohort)

| **Luminal type** |  | **High mutation**  **(N)** | **Low mutation**  **(N)** | **p value** |
| --- | --- | --- | --- | --- |
| Grade | 1 / 2 | 16 | 199 | 0.60 |
|  | 3 | 8 | 67 |  |
| AJCC T | T 1/2 | 37 | 468 | 0.09 |
|  | T 3/4 | 11 | 75 |  |
| pStage | Stage I / II | 32 | 391 | 0.28 |
|  | Stage III / IV | 15 | 129 |  |

| **Basal type** |  | **High mutation**  (N) | **Low mutation**  (N) | **p value** |
| --- | --- | --- | --- | --- |
| Grade | 1 / 2 | 0 | 3 | 0.45 |
|  | 3 | 14 | 51 |  |
| AJCC T | T 1/2 | 25 | 90 | 0.48 |
|  | T 3/4 | 5 | 12 |  |
| pStage | Stage I / II | 26 | 84 | 0.45 |
|  | Stage III / IV | 3 | 16 |  |

| **HER2 type** |  | **High mutation**  (N) | **Low mutation**  (N) | **p value** |
| --- | --- | --- | --- | --- |
| Grade | 1 / 2 | 5 | 3 | 0.06 |
|  | 3 | 3 | 15 |  |
| AJCC T | T 1/2 | 16 | 40 | 0.48 |
|  | T 3/4 | 5 | 6 |  |
| pStage | Stage I / II | 17 | 28 | 0.07 |
|  | Stage III / IV | 3 | 17 |  |

AJCC, American Joint Committee for Cancer

Supplementary Table S4: Validation cohort patient characteristics

|  |  | **High mutation**  (N) | **Low mutation**  (N) | **p value** |
| --- | --- | --- | --- | --- |
| Age | ≥ 50 | 161 | 1296 | 0.79 |
|  | < 50 | 42 | 360 |  |
| AJCC T | T 1/2 | 190 | 1577 | 0.73 |
|  | T 3/4 | 11 | 81 |  |
| AJCC N | N 0 | 116 | 851 | 0.13 |
|  | N + | 87 | 805 |  |
| pStage | Stage 1/2 | 125 | 1111 | 0.87 |
|  | Stage 3/4 | 11 | 109 |  |
| ER | - | 60 | 374 | 0.03 |
|  | + | 143 | 1282 |  |
| PR | - | 108 | 761 | 0.052 |
|  | + | 95 | 895 |  |
| HER2 | - | 175 | 1455 | 0.50 |
|  | + | 28 | 201 |  |
| TNBC | No | 144 | 1299 | 0.02 |
|  | Yes | 59 | 357 |  |
| *MKI67* | High | 120 | 809 | < 0.01 |
|  | Low | 83 | 847 |  |

AJCC, American Joint Committee for Cancer; ER, estrogen receptor; PR, progesterone receptor; TNBC, triple negative breast cancer
